# Supplementary material for: Benchmarking of eight recurrent neural network variants for breath phase and adventitious sound detection on a self-developed open-access lung sound database—HF_Lung_V1
Source: PLoS One. 2021 Jul 1;16(7):e0254134. doi: 10.1371/journal.pone.0254134 (PMC8248710; doi:10.1371/journal.pone.0254134)
Supplement: S1 Table — (DOCX) [file pone.0254134.s001.docx]

**S1 Table**

| Abbreviation | Definition |  | Abbreviation | Definition |
| --- | --- | --- | --- | --- |
| AI | artificial intelligence |  | **LSTM** | long short-term memory |
| AUC | area under the receiver operating characteristic curve |  | **MAL** | midaxillary line |
| BiGRU | bidirectional gated recurrent unit |  | **MAPE** | mean absolute percentage error |
| BiLSTM | bidirectional long short-term memory |  | **MCL** | midclavicular line |
| BMI | body mass index |  | **MFCCs** | mel-frequency cepstral coefficients |
| C | continuous adventitious sound event/label |  | **PPV** | positive predictive value |
| CAS(s) | continuous adventitious sound(s) |  | **R** | rhonchus event/label |
| CNN | convolutional neural network |  | **RCC** | respiratory care center |
| COVID-19 | coronavirus disease 2019 |  | **RCW** | respiratory care ward |
| D | discontinuous adventitious sound event/label |  | **RNN** | recurrent neural network |
| DAS(s) | discontinuous adventitious sound(s) |  | **ROC** | receiver operating characteristic curve |
| E | exhalation event/label |  | **S** | stridor event/label |
| Faster R-CNN | faster region-based CNN |  | **SIMP** | simplified |
| FN | false negative |  | **SNR(s)** | signal-to-noise ratio(s) |
| FP | false positive |  | **STFT** | short-time Fourier transform |
| GRU | gated recurrent unit |  | **TN** | true negative |
| I | inhalation event/label |  | **TP** | true positive |
| ICBHI | International Conference on Biomedical Health Informatics |  | **TSECC** | Taiwan Smart Emergency and Critical Care |
| ICS | intercostal space |  | **TWCC** | Taiwan Computing Cloud |
| JI | Jaccard index |  | **W** | wheeze event/label |
