# Supplementary material for: Benchmarking of eight recurrent neural network variants for breath phase and adventitious sound detection on a self-developed open-access lung sound database—HF_Lung_V1
Source: PLoS One. 2021 Jul 1;16(7):e0254134. doi: 10.1371/journal.pone.0254134 (PMC8248710; doi:10.1371/journal.pone.0254134)
Supplement: S2 Table — (DOCX) [file pone.0254134.s002.docx]

**S2 Table**

| Models | n of trainable parameters | Accuracy | | PPV | | Sensitivity | | Specificity | | *F1* score | |
| --- | --- | --- | --- | --- | --- | --- | --- | --- | --- | --- | --- |
|  |  | Segment | Event | Segment | Event | Segment | Event | Segment | Event | Segment | Event |
|  |  | Detection | Detection | Detection | Detection | Detection | Detection | Detection | Detection | Detection | Detection |
| LSTM | 300,609 | 0.890 | NA | 0.781 | 0.890 | 0.701 | 0.664 | 0.944 | NA | 0.739 | 0.761 |
| GRU | 227,265 | 0.899 | NA | 0.801 | 0.904 | 0.726 | 0.696 | 0.948 | NA | 0.762 | 0.789 |
| BiLSTM | 732,225 | 0.906 | NA | 0.814 | 0.885 | 0.750 | 0.772 | 0.951 | NA | 0.781 | 0.840 |
| BiGRU | 552,769 | 0.916 | NA | 0.836 | 0.898 | 0.773 | 0.800 | 0.956 | NA | 0.803 | 0.862 |
| CNN-LSTM | 3,448,513 | 0.903 | NA | 0.809 | 0.898 | 0.747 | 0.730 | 0.948 | NA | 0.776 | 0.811 |
| CNN-GRU | 2,605,249 | 0.905 | NA | 0.804 | 0.906 | 0.765 | 0.742 | 0.945 | NA | 0.784 | 0.820 |
| CNN-BiLSTM | 6,959,809 | 0.914 | NA | 0.822 | 0.902 | 0.791 | 0.803 | 0.950 | NA | 0.806 | 0.863 |
| CNN-BiGRU | 5,240,513 | 0.914 | NA | 0.829 | 0.898 | 0.785 | 0.812 | 0.952 | NA | 0.806 | 0.862 |
| SIMP BiLSTM | 235,073 | 0.906 | NA | 0.817 | 0.882 | 0.743 | 0.773 | 0.952 | NA | 0.778 | 0.841 |
| SIMP BiGRU | 178,113 | 0.915 | NA | 0.837 | 0.894 | 0.769 | 0.803 | 0.957 | NA | 0.801 | 0.861 |
| SIMP CNN-BiLSTM | 3,382,977 | 0.912 | NA | 0.828 | 0.895 | 0.774 | 0.799 | 0.953 | NA | 0.800 | 0.858 |
| SIMP CNN-BiGRU | 2,556,097 | 0.913 | NA | 0.830 | 0.889 | 0.774 | 0.810 | 0.953 | NA | 0.801 | 0.859 |

SIMP means the number of trainable parameters is adjusted.
