# Supplementary material for: Benchmarking of eight recurrent neural network variants for breath phase and adventitious sound detection on a self-developed open-access lung sound database—HF_Lung_V1
Source: PLoS One. 2021 Jul 1;16(7):e0254134. doi: 10.1371/journal.pone.0254134 (PMC8248710; doi:10.1371/journal.pone.0254134)
Supplement: S3 Table — (DOCX) [file pone.0254134.s003.docx]

**S3 Table**

| Models | n of trainable parameters | Accuracy | | PPV | | Sensitivity | | Specificity | | *F1* score | |
| --- | --- | --- | --- | --- | --- | --- | --- | --- | --- | --- | --- |
|  |  | Segment | Event | Segment | Event | Segment | Event | Segment | Event | Segment | Event |
|  |  | Detection | Detection | Detection | Detection | Detection | Detection | Detection | Detection | Detection | Detection |
| LSTM | 300,609 | 0.855 | NA | 0.716 | 0.561 | 0.406 | 0.456 | 0.962 | NA | 0.518 | 0.570 |
| GRU | 227,265 | 0.868 | NA | 0.715 | 0.687 | 0.514 | 0.554 | 0.951 | NA | 0.598 | 0.656 |
| BiLSTM | 732,225 | 0.866 | NA | 0.739 | 0.630 | 0.469 | 0.532 | 0.961 | NA | 0.573 | 0.639 |
| BiGRU | 552,769 | 0.882 | NA | 0.772 | 0.713 | 0.548 | 0.617 | 0.962 | NA | 0.641 | 0.709 |
| CNN-LSTM | 3,448,513 | 0.864 | NA | 0.732 | 0.628 | 0.476 | 0.512 | 0.957 | NA | 0.577 | 0.621 |
| CNN-GRU | 2,605,249 | 0.863 | NA | 0.731 | 0.629 | 0.470 | 0.516 | 0.958 | NA | 0.572 | 0.620 |
| CNN-BiLSTM | 6,959,809 | 0.867 | NA | 0.729 | 0.677 | 0.520 | 0.557 | 0.952 | NA | 0.604 | 0.656 |
| CNN-BiGRU | 5,240,513 | 0.874 | NA | 0.747 | 0.693 | 0.533 | 0.600 | 0.956 | NA | 0.622 | 0.685 |
| SIMP BiLSTM | 235,073 | 0.864 | NA | 0.736 | 0.612 | 0.450 | 0.520 | 0.962 | NA | 0.558 | 0.624 |
| SIMP BiGRU | 178,113 | 0.878 | NA | 0.741 | 0.716 | 0.559 | 0.603 | 0.954 | NA | 0.637 | 0.700 |
| SIMP CNN-BiLSTM | 3,382,977 | 0.869 | NA | 0.737 | 0.667 | 0.513 | 0.569 | 0.955 | NA | 0.604 | 0.662 |
| SIMP CNN-BiGRU | 2,556,097 | 0.873 | NA | 0.736 | 0.697 | 0.543 | 0.598 | 0.952 | NA | 0.624 | 0.684 |

SIMP means the number of trainable parameters is adjusted.
