# Supplementary material for: Benchmarking of eight recurrent neural network variants for breath phase and adventitious sound detection on a self-developed open-access lung sound database—HF_Lung_V1
Source: PLoS One. 2021 Jul 1;16(7):e0254134. doi: 10.1371/journal.pone.0254134 (PMC8248710; doi:10.1371/journal.pone.0254134)
Supplement: S4 Table — (DOCX) [file pone.0254134.s004.docx]

**S4 Table**

| Models | n of trainable parameters | Accuracy | | PPV | | Sensitivity | | Specificity | | *F1* score | |
| --- | --- | --- | --- | --- | --- | --- | --- | --- | --- | --- | --- |
|  |  | Segment | Event | Segment | Event | Segment | Event | Segment | Event | Segment | Event |
|  |  | Detection | Detection | Detection | Detection | Detection | Detection | Detection | Detection | Detection | Detection |
| LSTM | 300,609 | 0.812 | NA | 0.554 | 0.120 | 0.087 | 0.095 | 0.983 | NA | 0.151 | 0.122 |
| GRU | 227,265 | 0.812 | NA | 0.529 | 0.217 | 0.160 | 0.153 | 0.966 | NA | 0.246 | 0.201 |
| BiLSTM | 732,225 | 0.815 | NA | 0.579 | 0.155 | 0.119 | 0.167 | 0.980 | NA | 0.198 | 0.191 |
| BiGRU | 552,769 | 0.818 | NA | 0.574 | 0.237 | 0.176 | 0.227 | 0.969 | NA | 0.269 | 0.256 |
| CNN-LSTM | 3,448,513 | 0.840 | NA | 0.676 | 0.475 | 0.341 | 0.329 | 0.960 | NA | 0.453 | 0.425 |
| CNN-GRU | 2,605,249 | 0.849 | NA | 0.689 | 0.556 | 0.411 | 0.402 | 0.955 | NA | 0.515 | 0.498 |
| CNN-BiLSTM | 6,959,809 | 0.844 | NA | 0.686 | 0.443 | 0.369 | 0.419 | 0.959 | NA | 0.479 | 0.464 |
| CNN-BiGRU | 5,240,513 | 0.851 | NA | 0.690 | 0.508 | 0.435 | 0.463 | 0.952 | NA | 0.533 | 0.516 |
| SIMP BiLSTM | 235,073 | 0.814 | NA | 0.560 | 0.152 | 0.121 | 0.148 | 0.977 | NA | 0.198 | 0.179 |
| SIMP BiGRU | 178,113 | 0.814 | NA | 0.546 | 0.202 | 0.162 | 0.178 | 0.968 | NA | 0.250 | 0.222 |
| SIMP CNN-BiLSTM | 3,382,977 | 0.848 | NA | 0.688 | 0.490 | 0.403 | 0.443 | 0.956 | NA | 0.508 | 0.502 |
| SIMP CNN-BiGRU | 2,556,097 | 0.851 | NA | 0.699 | 0.499 | 0.423 | 0.475 | 0.955 | NA | 0.526 | 0.515 |

SIMP means the number of trainable parameters is adjusted.
