# Supplementary material for: Benchmarking of eight recurrent neural network variants for breath phase and adventitious sound detection on a self-developed open-access lung sound database—HF_Lung_V1
Source: PLoS One. 2021 Jul 1;16(7):e0254134. doi: 10.1371/journal.pone.0254134 (PMC8248710; doi:10.1371/journal.pone.0254134)
Supplement: S5 Table — (DOCX) [file pone.0254134.s005.docx]

**S5 Table**

| Models | n of trainable parameters | Accuracy | | PPV | | Sensitivity | | Specificity | | *F1* score | |
| --- | --- | --- | --- | --- | --- | --- | --- | --- | --- | --- | --- |
|  |  | Segment | Event | Segment | Event | Segment | Event | Segment | Event | Segment | Event |
|  |  | Detection | Detection | Detection | Detection | Detection | Detection | Detection | Detection | Detection | Detection |
| LSTM | 300,609 | 0.800 | NA | 0.716 | 0.699 | 0.556 | 0.485 | 0.905 | NA | 0.626 | 0.591 |
| GRU | 227,265 | 0.805 | NA | 0.697 | 0.746 | 0.624 | 0.514 | 0.883 | NA | 0.659 | 0.625 |
| BiLSTM | 732,225 | 0.821 | NA | 0.713 | 0.755 | 0.681 | 0.609 | 0.881 | NA | 0.696 | 0.700 |
| BiGRU | 552,769 | 0.827 | NA | 0.727 | 0.765 | 0.681 | 0.638 | 0.889 | NA | 0.703 | 0.714 |
| CNN-LSTM | 3,448,513 | 0.813 | NA | 0.706 | 0.734 | 0.672 | 0.526 | 0.876 | NA | 0.688 | 0.644 |
| CNN-GRU | 2,605,249 | 0.815 | NA | 0.725 | 0.709 | 0.640 | 0.539 | 0.893 | NA | 0.680 | 0.646 |
| CNN-BiLSTM | 6,959,809 | 0.830 | NA | 0.742 | 0.741 | 0.685 | 0.633 | 0.895 | NA | 0.712 | 0.708 |
| CNN-BiGRU | 5,240,513 | 0.826 | NA | 0.731 | 0.718 | 0.683 | 0.626 | 0.889 | NA | 0.706 | 0.700 |
| SIMP BiLSTM | 235,073 | 0.815 | NA | 0.702 | 0.764 | 0.675 | 0.582 | 0.876 | NA | 0.688 | 0.689 |
| SIMP BiGRU | 178,113 | 0.824 | NA | 0.718 | 0.779 | 0.689 | 0.626 | 0.883 | NA | 0.703 | 0.713 |
| SIMP CNN-BiLSTM | 3,382,977 | 0.828 | NA | 0.751 | 0.727 | 0.662 | 0.624 | 0.902 | NA | 0.702 | 0.702 |
| SIMP CNN-BiGRU | 2,556,097 | 0.825 | NA | 0.741 | 0.712 | 0.663 | 0.622 | 0.896 | NA | 0.699 | 0.695 |

SIMP means the number of trainable parameters is adjusted.
