# Supplementary material for: Benchmarking of eight recurrent neural network variants for breath phase and adventitious sound detection on a self-developed open-access lung sound database—HF_Lung_V1
Source: PLoS One. 2021 Jul 1;16(7):e0254134. doi: 10.1371/journal.pone.0254134 (PMC8248710; doi:10.1371/journal.pone.0254134)
Supplement: Attachment — Submitted filename: Response Letter_20210603.docx [file pone.0254134.s007.docx]

**Responses to the editor**

**1. Please ensure that your manuscript meets PLOS ONE's style requirements, including those for file naming. The PLOS ONE style templates can be found at**

<https://journals.plos.org/plosone/s/file?id=wjVg/PLOSOne_formatting_sample_main_body.pdf> and

<https://journals.plos.org/plosone/s/file?id=ba62/PLOSOne_formatting_sample_title_authors_affiliations.pdf>

Ans: We have checked the style guidelines. We have readjusted the font size and font weight of the headings and subheadings. We have removed the funding information from the Acknowledgments.

**2. Thank you for stating in your Funding Statement:**

**'This study was partially funded by the Raising Children Medical Foundation, Taiwan (http://**[**http://www.raising.org.tw**](http://www.raising.org.tw/)**). The funders had no role in study design, data collection and analysis, decision to publish, or preparation of the manuscript.'**

**a. Please provide an amended statement that declares *all* the funding or sources of support (whether external or internal to your organization) received during this study, as detailed online in our guide for authors at http://journals.plos.org/plosone/s/submit-now**

**Please also include the statement “There was no additional external funding received for this study.” in your updated Funding Statement.**

**b. Please include your amended Funding Statement within your cover letter. We will change the online submission form on your behalf.**

Ans: We have amended the “Funding Statement” as follows: “**Raising Children Medical Foundation, Taiwan, fully funded all of the lung sound collection and contributed the recordings to Taiwan Society of Emergency and Critical Care Medicine. The Heroic Faith Medical Science Co. Ltd, Taipei, Taiwan, freely provided the lung sound recording device (HF-Type-1) for the study and fully sponsored the data labeling and deep learning model training. There was no additional external funding received for this study**.

This statement is also included in the cover letter.

**3. Thank you for stating the following in the Competing Interests section:**

**'The authors have declared that no competing interests exist.'**

**We note that one or more of the authors are employed by commercial companies: Heroic Faith Medical Science Co. Ltd , Avalanche Computing Inc and HCC Healthcare Group.**

**a. Please provide an amended Funding Statement declaring this commercial affiliation, as well as a statement regarding the Role of Funders in your study. If the funding organization did not play a role in the study design, data collection and analysis, decision to publish, or preparation of the manuscript and only provided financial support in the form of authors' salaries and/or research materials, please review your statements relating to the author contributions, and ensure you have specifically and accurately indicated the role(s) that these authors had in your study. You can update author roles in the Author Contributions section of the online submission form.**

**Please also include the following statement within your amended Funding Statement.**

**“The funder provided support in the form of salaries for authors [insert relevant initials], but did not have any additional role in the study design, data collection and analysis, decision to publish, or preparation of the manuscript. The specific roles of these authors are articulated in the ‘author contributions’ section.”**

**If your commercial affiliation did play a role in your study, please state and explain this role within your updated Funding Statement.**

**b. Please also provide an updated Competing Interests Statement declaring these commercial affiliations along with any other relevant declarations relating to employment, consultancy, patents, products in development, or marketed products, etc.**

**Within your Competing Interests Statement, please confirm that these commercial affiliations do not alter your adherence to all PLOS ONE policies on sharing data and materials by including the following statement: "This does not alter our adherence to  PLOS ONE policies on sharing data and materials.” (as detailed online in our guide for authors http://journals.plos.org/plosone/s/competing-interests) . If this adherence statement is not accurate and  there are restrictions on sharing of data and/or materials, please state these. Please note that we cannot proceed with consideration of your article until this information has been declared.**

**c. Please include both an updated Funding Statement and Competing Interests Statement in your cover letter. We will change the online submission form on your behalf.**

**Please know it is PLOS ONE policy for corresponding authors to declare, on behalf of all authors, all potential competing interests for the purposes of transparency. PLOS defines a competing interest as anything that interferes with, or could reasonably be perceived as interfering with, the full and objective presentation, peer review, editorial decision-making, or publication of research or non-research articles submitted to one of the journals. Competing interests can be financial or non-financial, professional, or personal. Competing interests can arise in relationship to an organization or another person. Please follow this link to our website for more details on competing interests:** [**http://journals.plos.org/plosone/s/competing-interests**](http://journals.plos.org/plosone/s/competing-interests)

Ans: We have revised the “Competing Interests” as follows: “**FSH, SRH, YRC, YCL, BFH, YLW, TLT and CTT are full-time employees and CJH, NJL, WLT and YTC are part-time employees of Heroic Faith Medical Science Co. Ltd.** **CWH and CHC are with Avalanche Computing Inc., whom Heroic Faith Medical Science Co. Ltd. commissioned to train the deep learning models. This does not alter our adherence to PLOS ONE policies on sharing data and materials.**

This statement is also included in the cover letter.

**4. Please include captions for your Supporting Information files at the end of your manuscript, and update any in-text citations to match accordingly. Please see our Supporting Information guidelines for more information:** [**http://journals.plos.org/plosone/s/supporting-information**](http://journals.plos.org/plosone/s/supporting-information)

Ans: We have added the captions of the Supporting information files at the end of the manuscript. The in-text citations are updated accordingly.

**Responses to the reviewers**

Reviewer #1:

1. **What does benchmarking means here? Are they combining all these 8 models to generate single model?**

Ans: Benchmarking means a process of measuring the performance of the 8 models in breath phase and adventitious respiratory sound detection based on the newly established database. The importance of benchmarking is to provide a baseline reference for the future studies.

The answer to the second question is “No”. We reported the performance of all the 8 models in the lung sound analysis. Because we also tried to compare the performance between the 8 models in this study.

2. **Why RNN? What’s so special in RNN that is not covered by other variants of deep learning as well as the conventional learning?**

Ans: Because the auscultatory signal is temporal and the labels are one dimensional. The action of breathing is periodic and rhythmic, and the mechanisms of inhalation, exhalation and adventitious sound generation are temporally associated. RNN is widely accepted to analyze temporal signals.

We have experimented some models using only CNN variants. However, we found RNN can help refine the prediction results given by CNN and get a more stable prediction results when the dataset is still not large enough.

A new paragraph is also added to explain why we used deep learning instead of traditional machine learning (line 73–79).

3. **What does “S” means? Seconds?**

Ans: Yes, “s” means second. According to some writing guidelines, such as the one provided by IEEE (https://www.ewh.ieee.org/soc/ias/pub-dept/abbreviation.pdf), “s“ is a preferred abbreviation for second in SI unit.

4. **The major point is: What is new in all these the authors proposed?**

Ans: The major aims of the present study are to establish the largest open-access lung sound database and to report the benchmark tests using 8 different RNN-based models. In addition, the performance between the 8 RNN-based models is also compared. The study aims are summarized from line 123 to line 130 in the revised manuscript.

We have also revised the sentences in the sections of “Introduction” and “Related work” and positioned our work compared to the others more clearly. We have explained that so far only few studies have reported the results of sound detection at the recording level in respiratory sound analysis based on private datasets (line 92–98 and line 133–150). Therefore, without our benchmarking results and the open-access database, there are no baseline reference to be compared to in the future studies. We expect the benchmark results and the open-access database presented in this paper can greatly contribute to the development of respiratory sound analysis methods in the future.

5. **Which variant of CNN? There are many variants of CNN has been proposed, for instance, 3-D, 2-D, hybrid CNN, etc. Your are requested to check the other CNN models of CNN to bring some novelty in your work and the following repository may help you in your current and future studies** [**https://github.com/mahmad00**](https://github.com/mahmad00)

Ans: The CNN in this study means a simple 2D CNN with two convolutional layers. The detailed architectures of the networks can be found in Fig 4 and Fig 5.

We believe that advanced CNN variants could have better performance in feature extraction, and actually we have already experimented some of them. However, the benchmark results presented in this paper is just to serve as the first anchor for the future studies. Additionally, we consider using the models in a smart device for real-time respiratory monitoring so computing cost is a concern when using some complicated CNN structures or deeper CNN layers. Therefore, we started the research from some combinations of simple CNN and RNN layers.

It is mentioned in the section of “Conclusion” that the state-of-the-art CNN layers can be investigated in the future (line 551–552) and the advantage of using region-based CNN variants can be maximized in lung sound analysis if the labels are expanded to two-dimensional bounding boxes on the spectrogram (line 552– 554).

Thank you for providing the materials for us to research in the future. We will keep finding the most efficient and best solution to automated respiratory sound analysis.

Reviewer #2:
**1. Subsection “3.6 Task definition and evaluation metrics” line 293 [4] clearly —> need a proper citation.**

Ans: We have revised the sentence. It is now in the line 347 in the revised manuscript.

**2. Line 433 “double triggering” —> “a double triggering”.**

Ans: Thanks for the suggestion. However, after googling “a double triggering”, we found double triggering is used as an uncountable term in most search results. Hence, we decided not to add an “a” in the text.

**3. It would be good to have a table that lists the abbreviations.**

Ans: Thanks for the suggestion. We have put a list of abbreviations in S1 Table 1 in the section of “Supplementary information”.

**4. The authors need to explain the eight recurrent neural network models.**

Ans: We have added some descriptions to briefly explain the eight recurrent neural network models (line 288–295) and references are cited.

**5. The title of subsection “3.2 Preprocessing”, “3.7 Hardware and software”, and “2.4 Data labeling” are badly placed in the text.**

Ans: Thank you for the kind reminder. We have adjusted the position. The final typesetting will be helped by the staff of PLOS ONE.

6. **The title of Table 1 is badly placed.**

Ans: Thank you for the kind reminder. We have adjusted the position. The final typesetting will be helped by the staff of PLOS ONE.

**7. The authors need to improve the quality of Figure 2, Figure 3, Figure 3, Figure 6, and Figure 7.**

Ans: The quality of these figures is readjusted and checked by PACE as suggested by the editor. However, the submission system automatically downgrades the quality for some reasons when generating the pdf file for reviewing. The quality of the figure looks fine if it is downloaded from the provided link. We will keep communicating with the staff of PLOS ONE to make sure the quality is good when the paper is published.

**8. The authors need to provide more details on the simplified version of the RNN-based models.**

Ans: We have added a new figure (Fig 5) to illustrate the structures of simplified convolutional RNN models. Corresponding explanation is also added from the line 307 to line 309 in the revised manuscript.

**9. “The filtered signals were then processed using the short-time Fourier transform (STFT)”, The authors need to explain the STFT method.**

Ans: We have cited a book (Cohen L. Time-frequency analysis: Prentice Hall PTR Englewood Cliffs, NJ; 1995) and two previous studies to help explain the STFT. The parameters used in STFT are clearly described from line 270 to line 272.

**10. The authors need to add a related work section to clearly position their work comparing to others.**

Ans: We have reorganized the article and added a “Related work” section (line 133 to line 150). We clearly stated that so far only few other studies have reported the results of sound detection at the recording level in the respiratory sound analysis, and the dataset used in these studies are not open to the public. Hence, the benchmark results and the open-access database can be the foundation for future studies.

Reviewer #3:

1. **In Introduction section, the drawbacks of each conventional technique should be described clearly.**

Ans: In the first paragraph of the “Introduction” section, the limitations of conventional auscultation are explained clearly (line 58–67).

We have also added descriptions (line 73–79) to explain the drawback of using conventional machine learning and the strength of using deep learning to research computerized respiratory sound analysis.

2. **Introduction needs to explain the main contributions of the work more clearly.**

Ans: We have revised the manuscript and explained the reasons why we conducted this study: (1) In regard to the amount of data used in the deep learning, the more the better (line 88–91), and (2) only few studies have reported the results of sound detection at the recording level in the respiratory sound analysis based on private datasets (line 92–101). We clearly stated the aims of the studies in the last paragraph of introduction section (line 122–130).

**3. The authors should emphasize the difference between other methods to clarify the position of this work further.**

Ans: We have added a new section “Related work” (line 132–150). In this section, we have clearly stated that most studies focused on only the sound classification tasks. Only few studies have reported the results of sound detection at the recording level in the respiratory sound analysis, albeit based on private datasets.

**4. The Wide ranges of applications need to be addressed in Introductions**

Ans: We have added some descriptions to explain the application of computerized respiratory sound analysis (line 67–72). It can not only overcome the limitations of conventional auscultation but facilitate a tele-auscultation system.

5. **The objective of the research should be clearly defined in the last paragraph of the introduction section.**

Ans: We have revised the “Introduction: section to clarify why we conducted this study (line 84–101). The aims of the study have been summarized in the last paragraph of the introduction section (line 122–130).

6. **Fig 6 quality should be increased**

Ans: The quality of the figure is readjusted and checked by PACE as suggested by the editor. However, the submission system automatically downgrades the quality for some reasons when generating the pdf file for reviewing. The quality of the figure looks fine if it is downloaded from the provided link. We will keep communicating with the staff of PLOS ONE to make sure the quality is good when the paper is published.

7. **The authors can cite the following references
   Early detection of diabetic retinopathy using PCA-firefly based deep learning model
   An ensemble based machine learning model for diabetic retinopathy classification
   Deep learning and medical image processing for coronavirus (COVID-19) pandemic: A survey**

Ans: We have added a new paragraph in the “Introduction” section to discuss the reasons of using deep learning (line 74–79). We mentioned that deep learning is widely used in combating COVID-19 (line 80–81), and we have cited the third reference.

The other two papers are not closely related to this study. However, these references provide useful information for developing a better deep learning model for respiratory sound analysis. We could cite them in our future publications.
